# 

**Published:** 1887-12

**Authors:** 


10 cts. a copy DECEMBER, 1887. $1.00 per year.

### HALL'S JOURNAL of HEALTH

ESTABLISHED 1854

OFFICE: 206 BROADWAY, EVENING POST BUILDING, Room 11. N. Y.

Entered as Second-class Matter at the Post-Office, New York.

